# The Microevolution and Epidemiology of *Staphylococcus aureus* Colonization during Atopic Eczema Disease Flare

**DOI:** 10.1016/j.jid.2017.09.023

**Published:** 2018-02

**Authors:** Catriona P. Harkins, Kerry A. Pettigrew, Katarina Oravcová, June Gardner, R.M. Ross Hearn, Debbie Rice, Alison E. Mather, Julian Parkhill, Sara J. Brown, Charlotte M. Proby, Matthew T.G. Holden

**Affiliations:** 1 School of Medicine, University of St Andrews, St Andrews, UK; 2 Department of Dermatology, Ninewells Hospital, Dundee, UK; 3 School of Medicine, University of Dundee, Dundee, UK; 4 Institute of Biodiversity, Animal Health and Comparative Medicine, College of Medical, Veterinary and Life Sciences, University of Glasgow, Glasgow, UK; 5 Scottish Children’s Research Network, MACH 2 Building, Level 5, Ninewells Hospital, Dundee, UK; 6 Department of Veterinary Medicine, University of Cambridge, Cambridge, UK; 7 The Wellcome Trust Sanger Institute, Wellcome Trust Genome Campus, Hinxton, Cambridge, UK; 8 Skin Research Group, Division of Cancer Research, School of Medicine, University of Dundee, UK

**Keywords:** AE, Atopic eczema, CC, clonal complex, ST, sequence type, SNP, single-nucleotide polymorphism

## Abstract

*Staphylococcus aureus* is an opportunistic pathogen and variable component of the human microbiota. A characteristic of atopic eczema (AE) is colonization by *S. aureus*, with exacerbations associated with an increased bacterial burden of the organism. Despite this, the origins and genetic diversity of *S. aureus* colonizing individual patients during AE disease flares is poorly understood. To examine the microevolution of *S. aureus* colonization, we deep sequenced *S. aureus* populations from nine children with moderate to severe AE and 18 non-atopic children asymptomatically carrying *S. aureus* nasally. Colonization by clonal *S. aureus* populations was observed in both AE patients and control participants, with all but one of the individuals carrying colonies belonging to a single sequence type. Phylogenetic analysis showed that disease flares were associated with the clonal expansion of the *S. aureus* population, occurring over a period of weeks to months. There was a significant difference in the genetic backgrounds of *S. aureus* colonizing AE cases versus controls (Fisher exact test, *P* = 0.03). Examination of intra-host genetic heterogeneity of the colonizing *S. aureus* populations identified evidence of within-host selection in the AE patients, with AE variants being potentially selectively advantageous for intracellular persistence and treatment resistance.

## Introduction

Clinical studies have shown a link between *Staphylococcus aureus* and the pathogenesis of atopic eczema (AE). Affected individuals are characteristically prone to colonization by this pathogen (Hauser et al., 1985, Leyden et al., 1974), with disease severity correlating to bacterial burden and number of colonized sites (Tauber et al., 2016).

There has been a shift toward understanding how cutaneous dysbiosis contributes to AE etiology (Chng et al., 2016, Kennedy et al., 2016). Metagenomic analysis has shown that changes in populations of microbial communities are significantly associated with disease activity in AE, and that increased in *S. aureus* is linked to increasing severity (Kong et al., 2012). Although providing a more holistic overview of microbial population structure in AE, current metagenomic approaches have thus far failed to resolve the fine-scale population dynamics of *S. aureus* in AE or the genetic changes occurring in the colonizing population of this versatile pathogen.

Whole-genome sequencing is a high-resolution genotyping tool that can be used to study within-host evolution and transmission. Deep-sequencing studies of *S. aureus* populations have shown heterogeneity arising within the host and the impact on the disease-causing potential of the population (Azarian et al., 2016, Paterson et al., 2015, Young et al., 2012). By using whole-genome sequencing to genetically characterize bacteria, it is possible to investigate how populations differentiate and adapt within a host during colonization and to reconstruct the evolutionary events shaping populations (Didelot et al., 2016). In this study, we deep sequenced *S. aureus* populations from children with AE, enabling us to investigate the microevolution of colonization during disease flare. From this, we have uncovered evidence of selection enriching for colonization by *S. aureus* of specific genetic backgrounds, as well as genetic diversification promoting the survival and persistence of these strains during colonization of AE patients.

## Results

### *S. aureus* colonization in cases and controls

Nine AE patients were recruited through Ninewells Hospital in Dundee, UK, and skin swabs were obtained from five body sites, including a nostril, two areas of inflamed eczema skin, and two separate areas of clinically unaffected skin. All were colonized by *S. aureus* at one or more sampled sites. Eighteen community *S. aureus* nasal carriers were selected from the larger control study population, from whom swabs were taken from a single nostril. Extra-nasal skin swab from control participants were in all instances negative for *S. aureus*. Control age matching was attempted, but age-appropriate carriers identified had a history of atopy and were therefore excluded on this basis (Table 1).

**Table 1:** Participant characteristics of atopic eczema cases and healthy nasal carriage controls

Phenotype	Atopic Eczema Cases	Nasal Carriage Controls
Total number	9	18
Age in years, mean (range)	1.4 (0.25–4)	6.6 (5–8)
Sex, n	4 males/5 females	9 males/9 females
EASI score, mean (range)^1^	24.4 (12.8–37)	N/A
Atopic disease, n		
Atopic eczema	9	0
Asthma	0	0
Hay fever	2	0
Food allergy	3	0
Other inflammatory skin disease	0	0

Abbreviations. EASI, Eczema Area Severity Index; N/A, not applicable.

1 This range excludes patient 5, who had locally severe disease only.

All cases had generalized moderate to severe eczema, the exception being patient 5, who had locally severe disease only (see Supplementary Table S1 online). Four of the nine cases (44%) were nasally colonized in addition to eczema-affected skin, and seven (78%) were also colonized on clinically unaffected skin. Bacterial burden varied across colonization sites within individuals and among individuals (see Supplementary Table S2 online). Where available, five colonies from the primary isolation plates were randomly selected per swabbed body site to provide representative sampling and detect co-colonization (Votintseva et al., 2014). In patients with low bacterial burden, fewer than five colonies were available for whole-genome sequencing. Eczema site subsampling was included to investigate *S. aureus* population heterogeneity within disease sites. We undertook whole-genome sequencing of 10–28 colonies per AE case, depending on recovered colony counts.

### AE patients are colonized with distinct clonal populations of *S. aureus*

**Figure 1:**
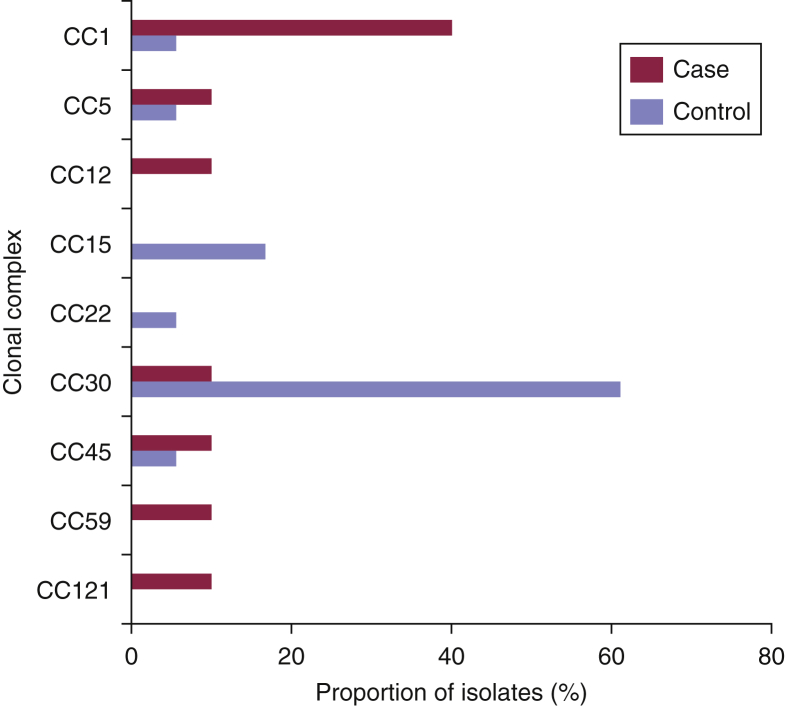
**Genetic diversity of *S. aureus* strains associated with AE flares.** The distribution of the clonal complex (CC) structure of *S. aureus* isolates from AE patients (n = 9) and nasal carriage control participants (n = 18). In the single patient in whom co-colonization (patient 8) was observed, both CCs identified in the colonizing population are represented. Skin and extra-nasal carriage was found in four AE patients (see Supplementary Table S2), three of whom were carrying CC1 isolates at both sites; one was carrying CC121 isolates (see Supplementary Table S3). Skin-only carriage isolates in AE patients (n = 5) were distributed across clonal backgrounds (see Supplementary Tables S2 and S3). AE, atopic eczema.

All controls and all but one case were colonized by clonal populations of *S. aureus* represented by a single sequence type (ST) defined by multilocus sequence typing (see Supplementary Table S3 online). The exception, patient 8, was co-colonized by two distinct STs. Comparison of the genetic backgrounds of the *S. aureus* colonizing populations in cases and controls showed different ST distributions (Figure 1). Eczema patients were more frequently colonized with STs belonging to clonal complex (CC) 1; four of the nine AE cases (44%) carried STs belonging to CC1 (ST1 and ST188), compared with a single control (6%; Fisher exact test, *P* = 0.03). Conversely, control participants were principally colonized with CC30; 11 of the 18 controls (61%) carried STs belonging to CC30 (ST30 and ST2889), compared with one case (11%; Fisher exact test, *P* = 0.02). This suggested that a subset of the *S. aureus* population is more frequently associated with colonization in AE. Overall, the proportions of CCs of isolates from cases and controls showed a significant difference in the distribution in CC of strains on the basis of disease status (Fisher exact test, *P* = 0.005).

### Clonal expansion of *S. aureus* populations in AE patients

The observed clonal populations in patients suggest that during disease flare there is a clonal expansion within the host, that is, the *S. aureus* growing on eczema skin originates from a small progenitor population within the individual. To provide a high-resolution view of the colonizing populations, we characterized the genomic diversity of the isolates and examined their phylogenetic relationships. As a measure of the relative within-host diversities of *S. aureus* populations, we examined the core genome of the sequenced isolates to identify single-nucleotide polymorphisms (SNPs). The levels of diversity we observed in both patients and control participants were comparable to those of previous studies examining the in-host population diversity of *S. aureus* during carriage (maximum pairwise SNP distance differentiating colonies from AE cases was 26 and in controls was 17) (Golubchik et al., 2013, Tong et al., 2015).

**Figure 2:**
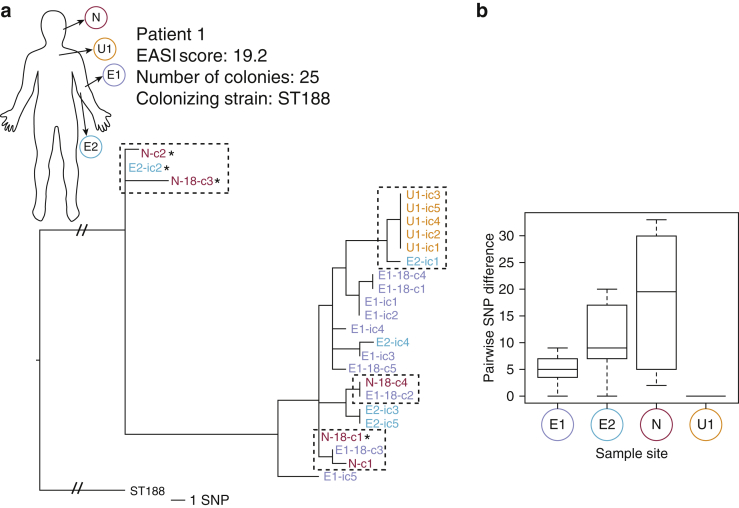
**Clonal expansion and self-transmission in patient 1.** (**a**) Maximum likelihood (ML) core SNPs tree illustrating genetic relationships of colonies across three body sites. Body diagram shows sampling site; branch label coloring corresponds to site from which colony was obtained. Perforated boxes indicate transmission between body sites. ^∗^β-lactamase carrying plasmid is absent. Branch labels: E, eczema (1 lateral/2 medial antecubital fossa); U, unaffected; N, nose; C, colony number from site; ic/-18 (initial colony preenrichment, 18 hour postenrichment). Tree rooted using ST188 reference. Scale bar = ∼1 SNP (not applicable to root branches with strikethrough). (**b**) Box plot comparing pairwise SNP difference between sequenced colonies from different body sites in a single patient. SNP, single-nucleotide polymorphism.

In AE cases where nasal colonization was detected, colonies derived from skin and nasal sites were interspersed throughout the phylogeny, suggesting exchange of *S. aureus* between sites rather than niche-specific populations (Figures 2a, and see Supplementary Figure S1 online). Comparison of the relative genetic diversities of populations from the different body sites in patient 1 showed a significant difference in the pairwise SNP distance separating colonies from different body sites (Kruskal-Wallis rank sum test, *P* = 3.9 × 10^–06^) (Figure 2b). Nasal isolates were distinguished by the greatest mean SNP distance per colony, suggesting that the *S. aureus* sampled from the patient’s nose had diversified over a longer period (Figure 2b). There was a significant difference in the pairwise SNP distance observed between nasal isolates and the eczema 1 subsampling site isolates (Wilcoxon-Mann-Whitney test, *P* = 0.015) but not compared with eczema 2 subsite colonies (Wilcoxon-Mann-Whitney test, *P* = 0.16). This shows that there is genetic and spatial diversity even within a single sampled site. Conversely, the absence of diversity in *S. aureus* from unaffected skin suggests very recent colonization of the site or potential variable replication rates influenced by the nutrient availability in the differing colonization environment (Figure 2a). The distribution of nasal isolates throughout the phylogeny, including basally, and intermingling with eczema isolates suggests that the nasal carriage represents a more established population and hence a potential source of *S. aureus*-colonizing diseased skin, therefore representing self-transmission.

**Figure 3:**
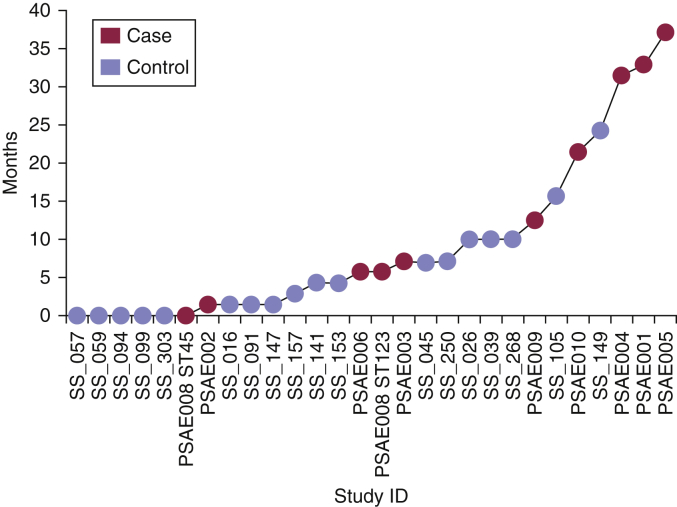
**Estimated age of the colonizing populations.** Time point to the most recent common ancestor of the population is presented in months and is calculated from half of the maximum pairwise SNP distance observed in the sampled populations. Both colony populations within patient 8 are included. ID, identification.

The *S. aureus* from AE cases and controls exhibited overlapping distributions of relative population diversities (patients, 0.1–2.6 SNPs/colony/individual; control participants, 0.0–3.4 SNPs/colony/individual), suggesting similar patterns of within-host diversification between the two groups (see Supplementary Table S4 online), even though the sampling in the controls was limited to a single niche. Using previously calculated mutation rates for *S. aureus,* we estimated the age of the sampled population for each participant. This suggested periods of 0 weeks to 37 months in patients and from less than a week to 24 months in control participants for the population to have diverged from a common ancestor (Figure 3).

### Evidence of adaption in the colonizing population

Diseased skin represents a physiologically distinct colonization environment compared with nasal epithelium, the primary carriage site of *S. aureus* in humans. We therefore hypothesized that the genomes of *S. aureus* from AE cases may exhibit evidence of environmental selection and adaption compared with controls. However, comparison of the distribution of mutation types between patients and control participants did not show a significant difference in frequency of nonsynonymous, synonymous, or intergenic SNPs (chi-square test, *P* = 0.74) (see Supplementary Figure S2a online). Examining the functional distribution of nonsynonymous mutations showed several gene categories differentially represented between study populations, but this was also not significant (Fisher exact test, *P* = 0.27) (see Supplementary Figure S2b).

**Figure 4:**
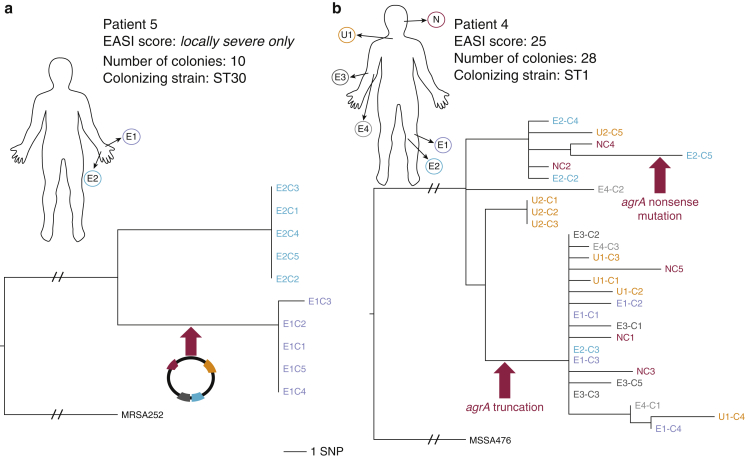
**Evidence of selection and genetic adaptation within the host.** (**a**) Evidence of plasmid dynamics and an evolutionary bottleneck. ML core SNPs tree illustrating genetic relationship of colonies from two spatially distinct positions in a single eczema site. Branch label marked with an arrow indicates point in phylogeny where a plasmid (backbone similar to SAP0194) carrying multiple metal resistance genes was gained in the *S. aureus* population. Tree rooted using MRSA252 reference. Branch labels: E1/3, lateral border; E2/4, medial border of single eczema site. SNP bar is indicated for scale (not applicable to root branches with strikethrough). (**b**) Convergent evolution of *agrA* mutants. ML core SNPs tree of colonies from five body sites. Branch labels marked with arrows indicate the point in phylogeny where homoplastic mutations in *agrA* are predicted to have occurred. Tree rooted using MSSA476 reference. EASI, Eczema Area Severity Index; ML, maximum likelihood; SNP, single-nucleotide polymorphism; ST, sequence type.

In the absence of signals of selection in AE cases at the cohort level, we looked for evidence of biologically and clinically relevant adaptation occurring in *S. aureus* at the patient level. Another source of variation in the *S. aureus* genome is in its content of mobile genetic elements, which constitute the accessory genome (Lindsay and Holden, 2006). Two instances of accessory genome diversification were identified in AE patients with loss/gain events of mobile genetic elements carrying genes potentially advantageous for environmental survival. Subsampling within a single site in patient 5 showed two distinct clades, separated by 26 SNPs, defining isolates derived from opposing borders of the site (Figure 4a). The absence of intervening variants suggested that in the sampled skin there had either been two separate acquisitions of the clade populations from a genetically closely related external population or a single acquisition and a selective sweep removing intermediate genetic variants, establishing spatially and genetically distinct populations. A 27-kilo base pair plasmid carrying heavy metal resistance determinants was differentially distributed between the clades. In patient 1, a plasmid carrying the β-lactamase gene, *blaZ,* was present in 21 of 25 colonies (Figure 2a), thereby rendering the colonizing population differentially sensitive to penicillin. In contrast, no such examples were seen in controls, despite similarly diverse carriage populations.

The impact of antimicrobials in shaping the colonizing populations was also evident in patient 1. All isolates from this patient contained two sequential point mutations in the gene encoding translation elongation factor G, *fusA.* The result was the amino acid change, L461K, which confers high-level fusidic acid resistance (Chen et al., 2010). The clinical history in this individual confirmed the use of this agent two months before sampling, which had not improved symptoms relating to eczema. The ancestor of this colonizing population is estimated to extensively predate this treatment (Figure 3), which would account for the lack of a therapeutic response, because colonization was by an already resistant population.

In addition to clinical interventions, we detected evidence of selection linked to survival in the host. In the *S. aureus* isolated from patient 4, two distinct mutations (see Supplementary Tables S5 and S6 online) ablating the expression of *agrA*, a global virulence regulator (Cheung et al., 2011, Fowler et al., 2004), were identified; one colony contained a nonsense mutation, and 18 colonies contained a frameshift mutation (Figure 4b). The phylogenetic context of these isolates showed they arose independently during the predicted period of colonization. The convergent evolution of mutations in this key regulator is evidence of a strong selective pressure favoring reduced virulence in this population.

## Discussion

Deep sequencing has allowed an unprecedented view of the microevolutionary changes occurring during carriage in AE, with evidence of selection for specific strain backgrounds colonizing AE skin. In-depth analysis of within-host genetic variation showed differences that affect both the pathogenic potential of the bacterial cells and their response to common clinical interventions.

Healthy nasal carriage controls exhibited a similar clonal population structure, indicating that the observed clonal expansion in AE was not a unique facet of the disease but rather a reflection of the natural colonization dynamics of *S. aureus*. In this regard, AE-affected skin represents an additional opportunistic niche for colonization, distinguished by an apparent selection for certain *S. aureus* lineages. A previous study of *S. aureus* strains from Korean AE patients did not find prevailing genotypes (Kim et al., 2009). However, the authors reported that one third of the isolates belonged to CC1, the same lineage differentially prevailing in our patients. This clonal lineage has recently been reported for its prevalence in AE patients in association with filaggrin mutations (Clausen et al., 2017). Nasal carriage studies by definition investigate niche-site colonization and repeatedly show CC30 and CC45 as the dominant lineages (Melles et al., 2004, Monecke et al., 2009). CC30 was the most prevalent lineage in our control participants, in agreement with these previous studies and more recently by Fleury et al. (2017). These findings raise the question of whether there are lineage-specific features making them more adept at colonization of the vastly differing cutaneous environments of the nasal versus inflamed eczema skin. This emphasizes an area where future studies must be directed.

The nose is often cited as the primary reservoir of *S. aureus* colonization associated with AE (Hoeger et al., 1992, Lomholt et al., 2005). Our study established evidence of self-transmission; however, in five of nine cases nasal colonization was notably absent, suggesting that the colonizing population was potentially derived from an extrinsic source. In these cases, transmission could have arisen from contact with a carrier, such as a family member or from the environment. It equally is possible that the point prevalence sampling did not capture transitory nasal carriage or that there was an unsampled reservoir as the source of self-transmission. An extended study assessing longitudinal colonization of individuals and their close contacts is needed to elucidate the origins of the flare-associated *S. aureus* population.

In this study, five AE cases had carriage populations estimated to have diverged from a common ancestor more than 12 months previously (Figure 3). In four of these cases, the predicted ancestral origins of the clonal colonizing population predates the age of the child. This suggests that the origins of the diversity observed in these individuals’ populations may have arisen during carriage within another host, with subsequent transmission of part of that population to the individual. Familial cross-transmission of *S. aureus* is likely to be highly relevant, and currently little is understood about this in the context of AE, although it has been shown that households are important reservoirs for transmission and diversification of *S. aureus* (Knox et al., 2015). In the future, serially sampling AE patients and their family members would enable us to assess the impact that selective sweeps and transmission bottlenecks have on shaping the diversity we observe in AE colonization.

From the most mature colonization populations, we uncovered clear evidence of selection shaping the populations, providing insights into mechanisms of bacterial persistence and other potential origins of the clonally expanded populations in these individuals. Data from two AE cases showed bacterial genetic responses linked with intracellular survival. The homoplastic mutations of *agrA* in patient 4 are strongly indicative of selection being exerted on the colonizing population. A recent in vitro study showed that *agr* mutants are able to internalize and persist within keratinocytes subverting host clearance, with *agr* mutants being recovered from keratinocytes at a frequency of over 58% (Soong et al., 2015). Independent mutations in *agrA* suggest that intracellular colonization and survival may have contributed to the persistence of *S. aureus* in patient 4. It is worth noting that intracellular populations are less penetrable by antimicrobial therapy and are therefore a potential cause of treatment failure. The *fusA* mutation in the *S. aureus* population in patient 1 who had previous exposure to fusidic acid is associated with a small colony variant phenotype (Norström et al., 2007). Small colony variants are phenotypic subpopulations occurring within a parent strain that are slow growing and show increased propensity to persist within host cells (Sendi and Proctor, 2009). Intracellular *S. aureus* has been reported in the context of both Darier disease and chronic rhinosinusitis as a cause of antimicrobial therapy failure (Hayes et al., 2015, von Eiff et al., 2001). Thus, we show two different mechanisms in separate cases that would support intracellular persistence and treatment resistance.

These findings highlight the therapeutic challenges of effectively eradicating *S. aureus* colonization in AE. During colonization there are potentially unrecognized genetic adaptations that render the population both insensitive and inaccessible to antimicrobial therapy, consequently prolonging proinflammatory interaction with the host. Longitudinal follow-up of the colonizing population for assessment of diversity, evidence of adaptation, and impact of therapies will advance our understanding of the relationship between *S. aureus* and disease activity. This is of particular relevance in children prone to repeated infective flares, for whom identifying and understanding genetic adaptation in the colonizing population may improve precision of treatment.

## Materials and Methods

### Recruitment and sampling

Prospective AE case and community control sampling studies received ethical approval from the Nottingham 1–East Midlands Research Ethics Committee (14/EM/1299) and East of Scotland Research Ethics service (15/ES/0153), respectively. Studies were conducted in accordance with the principles of the Declaration of Helsinki. Written informed parental consent and child assent were obtained before participation.

Cases were recruited at pediatric eczema clinics in Ninewells Hospital, Dundee, UK, between February and October 2015. Community control samples were obtained from 306 school children in Tayside and North Fife, UK, between November 2015 and February 2016. Case study inclusion criteria were age of 0–8 years and dermatologist-diagnosed moderate to severe AE. Exclusionary criteria were antimicrobials (systemic or topical) within the preceding 4 weeks, topical antiseptics within the preceding 2 weeks, or UV therapy within 3 months. The control participant study inclusion criterion was age of 0–12 years. Samples obtained from control study participants with parent-reported history of inflammatory skin disease, antibiotic therapy (<4 weeks previously), antiseptic therapy (<2 weeks), or UV therapy (<3 months) were not used as comparators in this study. Controls were selected from this study collection on the basis of age proximity to cases and no history of atopy or antimicrobial use, as per case criteria.

All participants were examined by an experienced dermatologist, and AE disease severity was scored using the Eczema Area Severity Index (Hanifin et al., 2001). Clinical history was obtained from patients during clinical review and from control participants via parental questionnaire. Cases were swabbed (Transtube Amies swab, Medical Wire, Corsham, England) from five sites including a single nostril, two areas of inflamed eczema, and two separate areas of unaffected skin. Eczema sites were subsampled, whereby a swab was taken from the lateral and medial border of the site, 4 cm apart. Controls were sampled from a single nostril and antecubital fossa.

### Bacterial isolation

Swabs were plated on Brilliance Staph 24 selective agar (Oxoid, UK) and incubated at 37 °C for 24 hours. Selective enrichment was also undertaken with swabs being used to inoculate 3 ml of Nutrient Broth with 7.5% NaCl (Oxoid, Basingstoke, UK) grown statically at 37 °C for 18 hours. 100 μl of broth was plated on selective agar, as described earlier. Colonies were then subcultured onto Brain Heart Infusion agar (Sigma Aldrich, Gillingham, UK) and confirmed as *S. aureus* by PCR detection of species-specific *femB* gene (Paterson et al., 2012).

### DNA extraction and whole-genome sequencing

Genomic DNA was extracted from overnight cultures of single colonies grown at 37 °C using Masterpure Gram Positive DNA purification kit (Epicentre, Cambridge, UK) as per the manufacturer’s protocol. DNA libraries were prepared using Nextera XT Library Preparation Kit (Illumina, Cambridge, UK) and quantified using Qubit High Sensitivity assay (LifeTechnologies, Paisley, UK) and Agilent Bioanalyser (Agilent, Stockport, UK). Libraries were normalized, pooled, and sequenced as paired-end reads on a MiSeq Genome Sequencer (Illumina).

### Bioinformatic and statistical analyses

Fastq files from MiSeq sequencing (see Supplementary Table S7 online) were assembled de novo with Velvet (Zerbino and Birney, 2008). Multilocus sequence types were predicted from sequence reads using SRST2 (Inouye et al., 2014). To identify SNPs, sequence reads were aligned to a reference genome of the same clonal complex (see Supplementary Table S8 online) using SMALT (http://www.sanger.ac.uk/science/tools/smalt-0). The default mapping parameters and SNP filtering were as previously described by (Hsu et al., 2015). Where an appropriate reference was unavailable, a de novo assembly from the participants’ samples was used for mapping; typically this was the assembly with the lowest number of contigs derived from the highest number of reads. Accessory genome regions (see Supplementary Table S9 online) were identified in the reference chromosomes using Artemis Comparison Tool (Carver et al., 2008) to compare pairwise BLASTN (Altschul et al., 1990) comparisons of reference genome sequences. Accessory regions were then masked from SNP alignments. The remaining core genome SNPs were individually curated by inspection of BAM files in Artemis to exclude false positives (see Supplementary Table S10 online); the subsequent SNPs were then used to construct maximum likelihood phylogenies with RAxML (Stamatakis, 2006). Indels were identified using GATK (https://software.broadinstitute.org/gatk/). Each participant’s reads were re-mapped to the de novo assembly, and GATK was used to identify indels compared with the patient reference sequence. Indels were curated by manual inspection of BAM files in Artemis (Rutherford et al., 2000).

The relative diversity in each participant’s colony population was calculated by dividing the number of core genome SNPs per sequenced colony per individual. Temporal calculation for the age of the *S. aureus* populations was based on half the maximum pairwise core SNP distance, and base substitution rates were derived from analysis of the major *S. aureus* lineages as described by Uhlemann et al., 2014. A rate of 1.6 × 10^–6^ SNPs/site/year was chosen as a median between published ranges. For each reference chromosome used, an expected base substitution rate per month was calculated based on the size of their respective core genomes. Functional classification of genes was conducted on the MSSA476 reference genome (Holden et al., 2004), using the previously described classification scheme in Gram-positive organisms (Weinert et al., 2015). All comparative statistics were performed as two-tailed tests using R software version 3.3.1 (R Core Team, 2016).

### Data access

Short reads for all sequenced isolates have been submitted to the European Nucleotide Archive (http://www.ebi.ac.uk/ena/) under project accession PRJEB20148.

## ORCIDs

Catriona P Harkins: http://orcid.org/0000-0002-9099-7291

Kerry A Pettigrew: http://orcid.org/0000-0002-6027-0462

Katarina Oravcová: http://orcid.org/0000-0001-5930-6803

Julian Parkhill: http://orcid.org/0000-0002-7069-5958

Sara J Brown: http://orcid.org/0000-0002-3232-5251

Matthew TG Holden: http://orcid.org/0000-0002-4958-2166

## Conflict of Interest

The authors state no conflict of interest.
