# Supplementary material for: The Microevolution and Epidemiology of Staphylococcus aureus Colonization during Atopic Eczema Disease Flare
Source: J Invest Dermatol. 2018 Feb;138(2):336–43. doi: 10.1016/j.jid.2017.09.023 (PMC5780352; doi:10.1016/j.jid.2017.09.023)
Supplement: Supplementary Figures S1 and S2 [file mmc2.pdf]

Figure S1

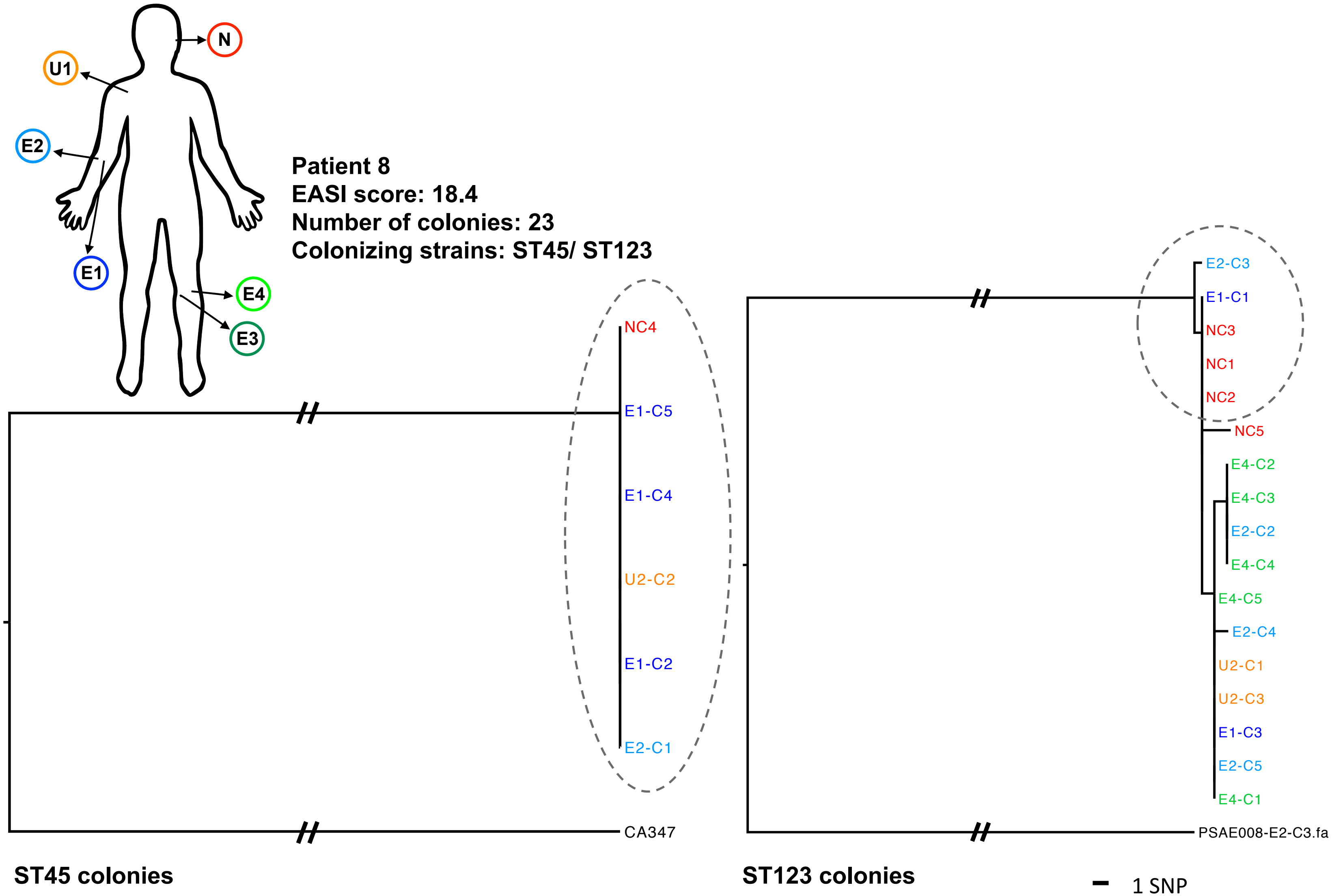
N
U1
E2
E1
E4
E3
Patient 8
EASI score: 18.4
Number of colonies: 23
Colonizing strains: ST45/ ST123
NC4
E1-C5
E1-C4
U2-C2
E1-C2
E2-C1
CA347
ST45 colonies
E2-C3
E1-C1
NC3
NC1
NC2
NC5
E4-C2
E4-C3
E2-C2
E4-C4
E4-C5
E2-C4
U2-C1
U2-C3
E1-C3
E2-C5
E4-C1
PSAE008-E2-C3.fa
ST123 colonies
1 SNP

Figure S1 Self-transmission and co-colonization.

Colonization by two distinct STs in Patient 8 (study ID PSAE008). Maximum likelihood core SNP tree of colonies from 4 body sites. Both strains are present nasally and extra-nasally. Branch label coloring corresponds to body site, marked on diagram. Perforated lines indicate overlapping spread between body sites. ST45 colonies are genetically identical. ST45 colony tree routed using CA347 reference; ST123 colony tree rooted used self-reference of E2-C3 colony assembly. Branch labels: E1/ 3- lateral border of single eczema site. E2/4- medial border of single eczema site. SNP bar is indicated for scale (not applicable to root branches with strikethrough).

Figure S2

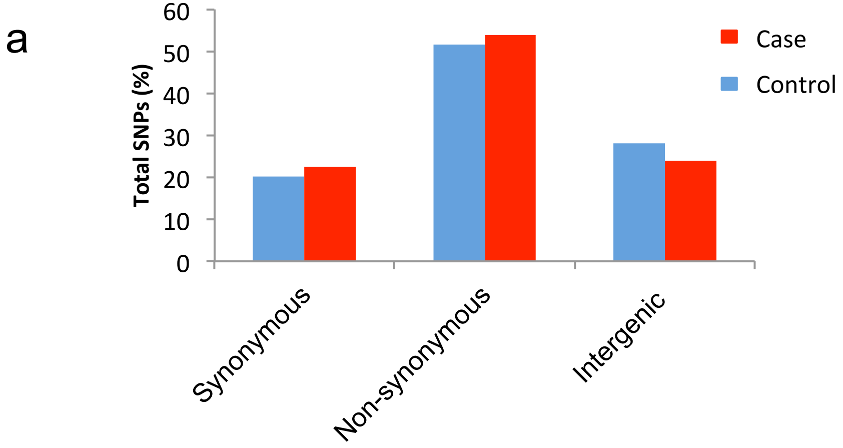
a
Total SNPs (%)
60
50
40
30
20
10
0
Synonymous
Non-synonymous
Intergenic
Case
Control

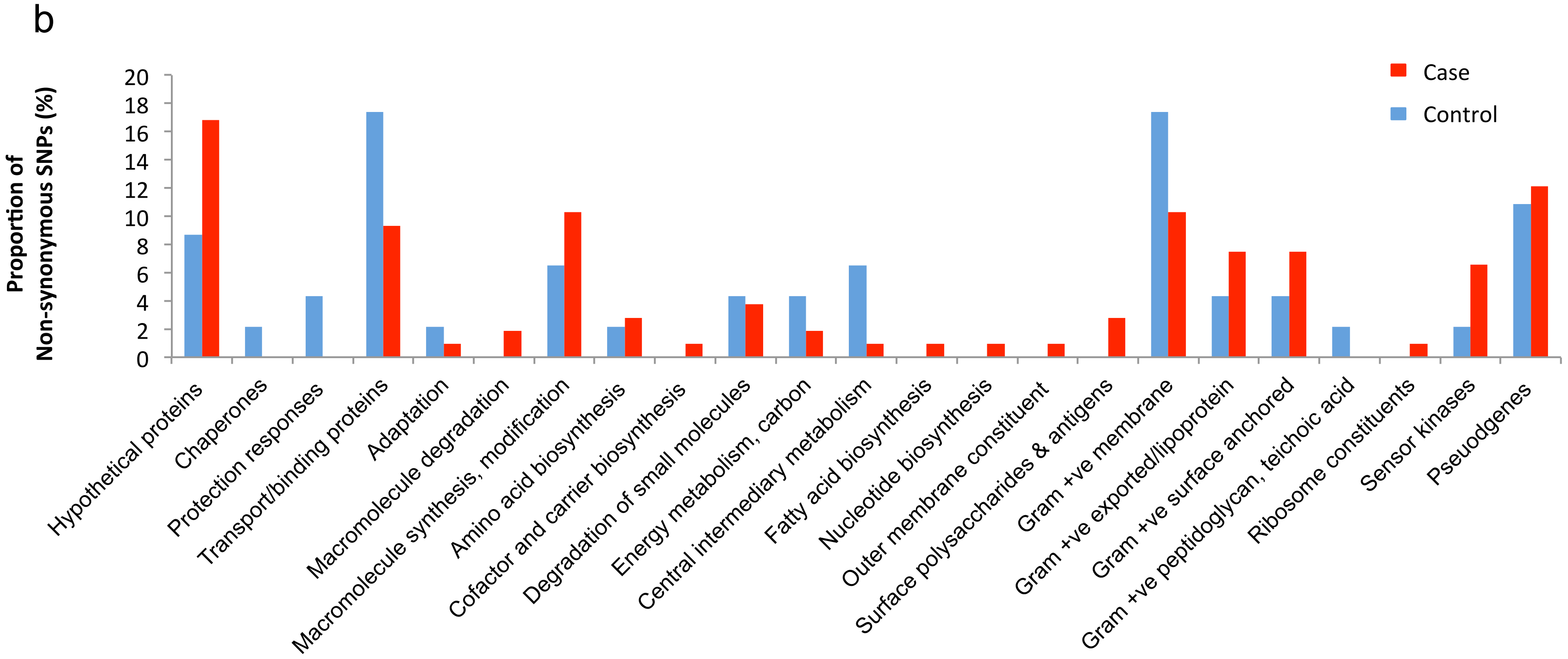
b
Proportion of
Non-synonymous SNPs (%)
20
18
16
14
12
10
8
6
4
2
0
Case
Control
Hypothetical proteins
Chaperones
Protection responses
Transport/binding proteins
Adaptation
Macromolecule degradation
Macromolecule synthesis, modification
Amino acid biosynthesis
Cofactor and carrier biosynthesis
Degradation of small molecules
Energy metabolism, carbon
Central intermediary metabolism
Fatty acid biosynthesis
Nucleotide biosynthesis
Outer membrane constituent
Surface polysaccharides & antigens
Gram +ve membrane
Gram +ve exported/lipoprotein
Gram +ve surface anchored
Gram +ve peptidoglycan, teichoic acid
Ribosome constituents
Sensor kinases
Pseudogenes

Figure S2 Distribution of SNPs by type and functional gene classification

(a) Distribution of SNP types occurring between cases and controls (total number of SNPs =285). (b) Distribution of non-synonymous base substitutions by functional gene class (n= 152) across the *S. aureus* genome.
